# Current News

**Published:** 1896-12

**Authors:** 


### Current News.

### NORTHEASTERN DENTAL ASSOCIATION.

The Second Annual Meeting of the Northeastern Dental Association was held at Springfield, Mass., October 21, 1896. About one hundred members were present. This Association was formed last year at Worcester, by the union of two associations that had occupied the field for many years. These were the Connecticut Valley and New England Dental Associations.

Three papers were given at the afternoon session, one by Dr. P. W. Moriarity, of Boston, on "Fractures of the Jaw," one by Dr. A. J. Flanagan, on "College Administration," and one on "Cataphoresis in Therapeutics," by Dr. Henry E. Waite, of New York City.

The following officers were chosen at the afternoon session: President, Dr. James H. Daly, of Boston; Vice-Presidents, Drs. D. B. Ingalls, of Clinton, and George A. Maxfield, of Holyoke; Secretary, Dr. Edgar O. Kinsman, of Cambridge; Assistant Secretary, Dr. A. J. Cutting, of Southington, Conn.; Treasurer, Dr. George A. Young, of Concord, N. H.; Librarian, Dr. George F. Cheney, of St. Johnsburg, Vt.; Editor, Dr. C. W. Strang, of Bridgeport, Conn.

The retiring president, Dr. James McManus, of Hartford, delivered the annual address at the evening session. He referred briefly to the formation of the Association, and urged the importance of loyalty to the State, district, and national societies. He spoke of the useful work of the old societies, and showed the importance of sustaining the new society,—that has for its field New England. The help from the society to the member should be practical, theoretical, scientific, and social. After the president's address, Dr. Arthur J. Wolff, of Hartford, Conn., bacteriologist to the Hartford Board of Health, gave an interesting illustrated lecture on "Bacteria." The address was a very profitable one from a scientific point, treating, as it did, not only of general principles, but of special forms of germ growth.

GEO. A. MAXFIELD, D.D.S.,
*Secretary.*

---

### NEW JERSEY STATE DENTAL SOCIETY.

AT the Twenty-sixth Annual Meeting of the New Jersey State Dental Society, held at Asbury Park, N. J., July 29–31, inclusive, the following-named officers were elected: President, Harvey Iredell, D.D.S., New Brunswick, N. J.; Vice-President, J. L. Crater, D.D.S., Orange, N. J.; Secretary, Charles A. Meeker, D.D.S., Newark, N. J.; Treasurer, Geo. C. Brown, D.D.S., Elizabeth, N. J.

CHAS. A. MEEKER,
*Secretary.*

